# Early and late results of open surgical and endovascular treatment of infrarenal abdominal aortic aneurysms, selected according to surgical risk

**DOI:** 10.1590/1677-5449.200024

**Published:** 2021-11-29

**Authors:** José A. Torres Hernández, Mercedes Sánchez-Barba, Jesús García-Alonso, Magdalena Sancho, José R. González-Porras, Francisco Santiago Lozano Sanchez

**Affiliations:** 1 Universidad de Salamanca – USAL, Instituto de Investigación Biomédica de Salamanca – IBSAL, Hospital Universitario de Salamanca, Servicio de Angiología y Cirugía Vascular, Salamanca, Spain.; 2 Universidad de Salamanca – USAL, Servicio de Estadística, Salamanca, Spain.; 3 Universidad de Salamanca – USAL, Instituto de Investigación Biomédica de Salamanca – IBSAL, Hospital Universitario de Salamanca, Servicio de Radiología, Salamanca, Spain.; 4 Universidad de Salamanca – USAL, Instituto de Investigación Biomédica de Salamanca – IBSAL, Hospital Universitario de Salamanca, Servicio de Anatomía Patológica, Salamanca, Spain.; 5 Universidad de Salamanca – USAL, Instituto de Investigación Biomédica de Salamanca – IBSAL, Hospital Universitario de Salamanca, Servicio de Hematología, Salamanca, Spain.

**Keywords:** abdominal aortic aneurysm, aortic open surgery, endovascular aortic repair, aneurisma da aorta abdominal, cirurgia aberta da aorta, reparo endovascular da aorta

## Abstract

**Background:**

Open surgical repair (OSR) and endovascular aneurysm repair (EVAR) surgery are alternative treatments for infrarenal abdominal aortic aneurysm (IRAAA).

**Objectives:**

To compare OSR and EVAR for the treatment of IRAAA.

**Methods:**

119 patients with IRAAA were electively operated by the same surgeon between January 1, 2006 and December 31, 2015, following selection for OSR or EVAR according to surgical risk. Complications, reinterventions, failures, and early and late mortality were analyzed.

**Results:**

63 OSR and 56 EVAR patients were analyzed. They were similar in terms of age (70 years), gender (92% men), and average diameter of IRAAA (6.5 cm), but with different comorbidities, surgical risk, and anatomy. EVAR was better than OSR regarding time in the operating theatre (177.5 *vs.* 233.3 minutes), need for transfusion (25 vs. 73%), and length of stay in ICU (1.3 *vs.* 3.3 days) and hospital (8.1 *vs.* 11.1 days). OSR allowed more associated procedures to be conducted simultaneously (19.0 *vs.* 1.8%). There were no significant differences between the groups with respect to complications (25.4 *vs.* 25.1%), reinterventions (3.2 *vs.* 5.2%), or early mortality (1.6 *vs.* 0%). During follow-up, OSR was associated with fewer revisions (3.13 *vs.* 4.21), angio-CTs (0.22 *vs.* 3.23), complications (6.4 *vs.* 37.5%), reinterventions (3.2 *vs.* 23.2%), and failures (1.6 *vs.* 10.7%), and had better survival (78.2 *vs.* 63.2%).

**Conclusions:**

Correct selection of patients achieves excellent results because it avoids OSR in patients at high risk and avoids EVAR in patients with high anatomical complexity, achieving similar results in the perioperative period, but better results for OSR over the course of follow-up.

## Resumo

**Contexto:**

A cirurgia aberta (CA) e o reparo endovascular de aneurisma (REVA) são tratamentos alternativos para o aneurisma da aorta abdominal infrarrenal (AAAIR).

**Objetivos:**

Comparar CA e REVA no tratamento do AAAIR.

**Métodos:**

Foram incluídos 119 pacientes com AAAIR, operados eletivamente pelo mesmo cirurgião entre 1 de janeiro de 2006 e 31 de dezembro de 2015, após seleção para CA ou REVA de acordo com o risco cirúrgico. Complicações, reintervenções, falhas e mortalidade precoce e tardia foram analisadas.

**Resultados:**

Foram analisados 63 pacientes de CA e 56 de REVA, com semelhanças de idade (70 anos), sexo (92% homens) e diâmetro médio do AAAIR (6,5 cm), mas com diferentes comorbidades, riscos cirúrgicos e anatomias. O REVA foi melhor que a CA em relação ao tempo na sala de cirurgia (177,5 vs. 233,3 minutos), necessidade de transfusão (25 vs. 73%) e tempo de permanência na unidade de terapia intensiva (1,3 vs. 3,3 dias) e no hospital (8,1 vs. 11,1 dias). A CA permitiu que mais procedimentos associados fossem realizados simultaneamente (19,0 vs. 1,8%). Não houve diferenças significativas entre os grupos em relação a complicações (25,4 vs. 25,1%), reintervenções (3,2 vs. 5,2%) e mortalidade precoce (1,6 vs. 0%). Durante o acompanhamento, a CA apresentou menos revisões (3,13 vs. 4,21), angiotomografias (0,22 vs. 3,23), complicações (6,4 vs. 37,5%), reintervenções (3,2 vs. 23,2%) e falhas (1,6 vs. 10,7%), além de ter melhor sobrevida (78,2 vs. 63,2%).

**Conclusões:**

A seleção correta dos pacientes proporciona excelentes resultados porque evita pacientes com alto risco para CA e com complexidade anatômica para REVA. Os resultados são semelhantes no período perioperatório, mas melhores para CA durante o acompanhamento.

## INTRODUCTION

Infrarenal abdominal aortic aneurysms (IRAAAs) are relatively frequent in men, those over 65 years of age, the hypertensive, and smokers, and have a low incidence in diabetics.^1^ This is an asymptomatic disease, but a percentage of IRAAAs can grow until rupture occurs and the patient dies. IRAAAs that require treatment can be resolved in two ways. The first is to replace the aneurysm with a prosthetic graft using open surgical repair (OSR). This method has undergone hardly any technical changes for several decades and experienced teams achieve very good results.^2^ The second, more recently introduced method, is exclusion of the aneurysm through the endovascular aneurysm repair (EVAR) technique, which, being less aggressive, has quickly become an established procedure.^3^ Prospective studies comparing the two treatment methods report that EVAR results in lower perioperative morbidity and mortality, but that, over a period of years, this advantage is lost, with patients presenting similar survival rates, but higher rates of complications, repeat operations, and aneurysm-related mortality.^4^

According to the recommendations of therapeutic guidelines,^5^^,^ 6 EVAR is the technique of choice for patients with optimal anatomy but who are considered to be at high risk from OSR. In recent years, many groups have been carrying it out, to the extent that in the USA it is provided to almost 80% of patients.^7^

In this context, the present study analyzes the clinical results of both techniques when patients are selected for one of the two therapeutic modalities based on their age and medical history (risks), following current clinical criteria. The ultimate objective is to assess the morbidity and mortality during the perioperative period and throughout follow-up in order to demonstrate that the two techniques are complementary, rather than exclusive.

## MATERIAL AND METHODS

A retrospective study of a cohort of 119 patients with asymptomatic IRAAA, electively operated on by the Angiology and Vascular Surgery Service of the Hospital Universitario de Salamanca between 1 January 2006 and 31 December 2015, by a team whose principal surgeon, who had experience in both techniques, was always the same person.

**Figure 1:**
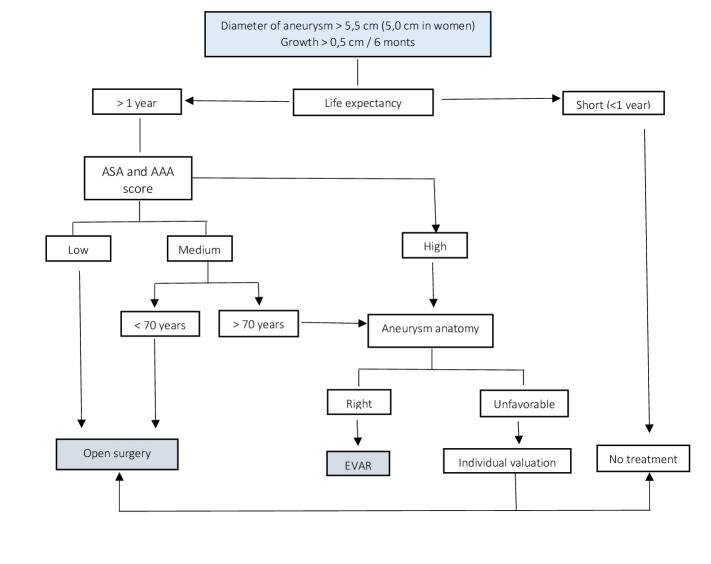
Algorithm that determines the technique to be used.

In a systematic manner, patients were selected for either therapeutic modality in clinical sessions at the service based on the balance of risk and benefit from OSR or EVAR according to age, comorbidities, existence of a hostile abdomen, anatomy of the IRAAA (Table 1), possibility of treating another serious retroperitoneal pathology during the same operative session, the need for rapid recovery in order to treat other diseases, or according to the patient’s choice (Figure 1). The prosthesis used in OSR was always one made of polyester impregnated in gelatin (Dacron, Unigraf, B Braun®). In the case of EVAR, a prosthesis made from low-porosity polyester with suprarenal fixation was used, the Talent® model (Medtronic Ibérica, S.A.) up to the end of 2009 and then Endurant® and Endurant II® models (Medtronic Ibérica, S.A.) since 2010.

**Table 1:** Contraindications for EVAR, according to characteristics of the aortic neck.

Diameter	< 17 mm or > 32 mm
Angle	> 60º
Length	< 15 mm (up to 10 mm exceptionally)
Thrombus	> 50% of perimeter
Calcification	> 50% of perimeter
	> 3 mm in the first 10 mm

Source: Gómez Palonés et al.^6^

Data were collected after reviewing the clinical history and imaging tests from the first admission and during follow-up. Demographic parameters, vascular risk factors, medical history, analytical determinations, ASA risk (American Society of Anesthesiologists), preoperative surgical risk assessed by applying the specific model for IRAAA published by Ambler et al.,^8^ and morphological parameters were evaluated. Several technical variables derived from the surgical activity, ICU and hospital stays, postoperative complications, reinterventions, failures, and short-term (30 days) and long-term (follow-up) mortality, as well as survival were analyzed.

Failures of the technique are defined as: 1) mortality related to the aneurysm or its treatment; 2) complications that had to be resolved by changing the therapeutic modality; and 3) when the aneurysm repressurized and was tending to grow, and when it was not feasible to resolve it due to technical impossibility or patient refusal.

The patients included in the study were consecutive. The institutional review board at the Facultad de Medicina de la Universidad de Salamanca approved this study (DOC-CIR/ODON-20012017) and waived the need for patient consent. No patients were excluded, because, except for two, patients were not lost from the study until their death. These two patients were contacted and they agreed to be reviewed and for a control angio-CT to be performed. In some deceased patients whose cause of death was not ascertained by other means, data collection was completed by telephone interview with their relatives.

Statistical analysis was performed with IBM SPSS version 23.0. Statistical tests were used as appropriate for the type of variable. Survival was analyzed by the Kaplan-Meier method and a Cox regression analysis was carried out to determine the factors that influenced appearance of postoperative complications and survival. The sample size was calculated with the GRANMO power calculator (Version 7.12 April 2012). The level of statistical significance was p < 0.05.

## RESULTS

During the study period, 502 patients were treated by IRAAA, 119 of whom were operated on as scheduled by a team with the same principal surgeon. Initially, 62 patients were selected for OSR and 57 for EVAR, but one patient in the latter group reconverted and was analyzed in the OSR group, implying an initial conversion rate of 1.75%. Thus, finally, 63 and 56 patients were included in the OSR and EVAR groups, respectively.

Table 2 shows the baseline characteristics of the patients in both groups. Patients were mostly males in the seventh decade of life, the distributions being the same for both groups. Among the risk factors, the only difference was that there were more smokers in the EVAR group (p < 0.025). There were more patients with a history of heart disease (p = 0.027), chronic renal failure (CRF) (p = 0.041), chronic obstructive pulmonary disease (COPD) (p < 0.001), and hostile abdomen (p < 0.001) in the EVAR group. Likewise, patients in the EVAR group had a greater ASA anesthetic risk (p < 0.001) and a higher preoperative surgical risk, although, when corrected for the therapeutic modality applied, this made the actual risk of surgery lower in the EVAR group (1.65 *vs.* 2.58).

**Table 2:** Baseline characteristics of the groups.

	**Open (n = 63)**	**EVAR (n = 56)**	**p value**
Demographics			
Age, years (mean ± SD, range)	72.2 ± 6.6 (59-84)	70.0 ± 6.9 (54-84)	NS
Male sex (n, %)	58 (92.1)	52 (92.9)	NS
White race (n, %)	63 (100)	56 (100)	NS
Cardiovascular risk factors (n, %)			
Arterial hypertension	50 (79.4)	42 (75.0)	NS
Diabetes mellitus	10 (15.9)	13 (23.2)	NS
Dyslipidemia	42 (66.7)	36 (64.3)	NS
Active smoking	43 (68.3)	48 (85.7)	0.025
Current history (n, %)			
Heart disease*	20 (31.7)	29 (51.8)	0.027
TIA/ictus	1 (1.6)	4 (7.1)	NS
Chronic kidney disease	3 (4.8)	9 (16.1)	0.041
COPD	4 (6.3)	22 (39.3)	0.001
PAD (lower extremities)	16 (25.4)	21 (37.5)	NS
Cancer	14 (22.2)	18 (32.1)	NS
Hostile abdomen	3 (4.8)	9 (16.1)	0.001
Surgical risk			
ASA class (n, %)			0.001
2	28 (44.4)	7 (12.5)	
3	32 (50.8)	42 (75.0)	
4	3 (4.8)	7 (12.5)	
AAA score (middle value)			
Basal	2.58	3.56	-
Corrected**	2.58	1.65	-
Morphological data			
Aneurysm diameter (cm, mean)	6.55	6.36	NS
Aortic neck (mm, mean)	18,92	26.91	0.001
Pathological aortic neck (n, %)	11 (17.5)	1 (1.8)	0.005
Neck angle > 60º (n, %)	6 (9.52)	0 (0.0)	0.037
Pathological iliac arteries (n, %)	8 (12.7)	0 (0.0)	0.006
Iliac aneurysm (n, %)	16 (25.4)	15 (26.8)	NS
Indications of the technique (n, %)			
Low risk	36 (57.1)	0 (0.0)	-
High risk	0 (0.0)	41 (73.2)	-
Associate another surgery	4 (6.5)	0 (0.0)	-
Inappropriate anatomy (EVAR)	19 (30.0)	0 (0.0)	-
Hostile abdomen	0 (0.0)	9 (16.0)	-
Fast recovery***	0 (0.0)	2 (3.6)	-
Reconversion	1 (1.6)	0 (0.0)	-
Patient choice	3 (4.8)	4 (7.2)	-

SD: Standard Deviation; NS: Not Significant (p > 0.05); TIA: Transient Ischemic Attack; COPD: Chronic Obstructive Pulmonary Disease; PAD: Peripheral Artery Disease; ASA: American Society of Anesthesiology (risk classification); AAA: Abdominal Aortic Aneurysms; EVAR: Endovascular Aneurysm Repair. AAA score.^8^

* Mostly ischemic heart disease;

** According to technique (open or EVAR);

*** Needed fast recovery for another treatment.

There were no differences in the mean diameter of the aneurysm (mean > 6 cm), or in the association with aneurysm in the iliac arteries. In contrast, the characteristics of the aortic neck and the iliac arteries were significantly worse in the OSR group. The indications for OSR were mostly considered low risk (57.1%) and high risk for EVAR (73.2%). In four cases of OSR, the decision was made to simultaneously resolve another serious retroperitoneal pathology: one was a case of an exeresis of a malignant fibrous histiocytoma and three were cases of nephrectomies due to renal carcinoma. In two cases of EVAR, the indication was to achieve a rapid recovery, and to treat a multiple myeloma in one case and a pulmonary adenocarcinoma in the other.

Table 3 shows the perioperative technical results. There were significantly better results for EVAR with respect to the duration of the surgical intervention (p < 0.001), the length of stay in the ICU (p = 0.007), the length of hospital stay (p = 0.02), the need for transfusion (p < 0.001), and the number of transfused red cell concentrates (p < 0.001). The advantage of OSR was that more associated surgeries were performed during the intervention than were done with EVAR (12 *vs.* 1) (p = 0.003). The associated procedures with OSR were four cases of cholecystectomies, three nephrectomies, an inguinal herniorrhaphy, one exeresis of a malignant fibrous histiocytoma, one lymphadenectomy, one femoral-popliteal bypass, and one endoprosthesis exclusion of a thoracic aortic aneurysm (TEVAR). In the EVAR group, the associated procedure was a TEVAR.

**Table 3:** Perioperative technical results by groups.

	**Open (n = 63)**	**EVAR (n = 56)**	**p-value**
Preoperative arteriography (n, %)	6 (9.5)	10 (17.9)	NS
Hypogastric embolization (n, %)	4 (6.3)	9 (16.1)	NS
Anesthesia (local-regional) (n, %)	0 (0)	53 (94.6)	0.001
Concomitant procedures (n, %)	12 (19.0)	1 (1.8)	0.003
Infrarenal clamp position (n, %)	52 (82.5)	-	-
Procedure time (minutes) (mean ± SD)	233.3±64.1	177.5±43.7	0.001
ICU stay (days) (mean ± SD)	3.35±5.86	1.29± 0.59	0.007
Length of stay (days) (mean ± SD)	11.1±8.47	8.07±4.85	0.02
Blood transfusion (n, %)	46 (73.0)	14 (25.0)	0.001
Red cells transfusion (n) (mean ± SD)	2.46±2.71	0.46±0.87	0.001
Serum creatinine (mg/dL) (mean ± SD)			
Pre-intervention	1.12±0.43	1.32±1.18	NS
Post-intervention (48 h)	0.97±0.62	1.21±1.34	NS
Prosthesis type (n, %)			
Straight	30 (47.6)	-	-
Bifurcated (bi-iliac)	14 (22.2)	-	-
Bifurcated (femoral)	19 (30.2)	-	-
Bifurcated endograft	-	28 (50)	-
Aorto-monoiliac endograft	-	28 (50)	-
Talent®	-	29 (51.8)	-
Endurant®	-	27 (48.2)	-

ICU: Intensive Care Unit; SD: Standard Deviation; NS: Not Significant (p > 0.05).

In the OSR group, an aorto-aortic graft was inserted in 47.6% of the cases, bifurcated to iliac arteries in 22.2%, and with anastomosis to at least one femoral graft in 30.2%. In the EVAR technique, a bifurcated stent was implanted in 50% and an aorto-monoiliac stent in the other 50%. Up to 2010, the Talent® model had been used in 29 cases, and thereafter the Endurant® or Endurant II® model was used in 27 cases. The implant was fixed in the external iliac artery in 13 cases (23.2%).

Table 4 shows the early and late results by group. No differences were found between groups in terms of complications, reinterventions, or 30-day mortality. Sixteen OSR patients and 14 EVAR patients presented some kind of complication. The most frequent complications in OSR patients were bleeding (12.7%), respiratory complications (9.5%), and renal failure (6.3%); with the EVAR technique the most frequent were bleeding (8.9%), respiratory complications (8.9%), urinary infection (7.1%), and cardiological complications (5.4%). Five patients were reoperated, two from the OSR group and three from the EVAR group. Only one patient died, a member of the OSR group, implying a 1.6% mortality rate in this group and a 0.84% rate in the entire series overall.

**Table 4:** Early and late postoperative results by groups.

	**Open (n = 63)**	**EVAR (n = 56)**	**p-value**
EARLY (30 days)			
No complication (n, %)	47 (74.6)	42 (75.0)	NS
Complications (n, %)	16 (25.4)	14 (25.0)	NS
Bleeding	8 (12.7)	5 (8.9)	NS
Renal insufficiency	4 (6.3)	2 (3.6)	NS
Respiratory insufficiency/pneumonia	6 (9.5)	5 (8.9)	NS
Cardiac/coronary insufficiency	1 (1.6)	3 (5.4)	NS
Urinary infection	2 (3.2)	4 (7.1)	NS
Wound infection	1 (1.6)	1 (1.8)	NS
Sepsis	1 (1.6)	0 (0.0)	NS
Other complications	2 (3.2)	2 (3.6)	NS
Reinterventions (n, %)	2 (3.2)	3 (5.2)	NS
Mortality (n, %)	1 (1.6)	0 (0)	NS
LATE (Follow-up)			
Years (median)	4.25	4.79	NS
Revisions (n) (mean ± SD)	3.13 ± 1.65	4.21 ± 2.52	0.007
TC control (n) (mean ± SD)	0.22 ± 0.58	3.23 ± 2.02	0.001
Complications (n, %) *	4 (6.4)	21 (37.5)	0.001
Anastomotic pseudoaneurysm	1 (1.6)	NA	
Incisional hernia	3 (4.8)	NA	
Acute renal failure	0 (0)	1 (1.8)	
Periprosthetic infection	0 (0)	1 (1.8)	
Branch thrombosis	0 (0)	4 (7.1)	
Rupture	0 (0)	2 (3.6)	
F-F bypass thrombosis	NA	6 (10.7)	
Endoleak	NA	14 (25.0)	
Types:			
1	NA	5 (8.9)	-
2	NA	12 (21.4)	-
3	NA	1 (1.8)	-
5	NA	2 (3.6)	-
Reinterventions (n, %)*	2 (3.2)	13 (23.2)	0.001
Incisional hernia repair	1 (1.6)	NA	
Femoral pseudoaneurysm repair	1 (1.6)	0 (0)	
Reconversion	0 (0)	3 (5.4)	
Thrombectomy	0 (0)	5 (8.9)	
Bypass	0 (0)	3 (5.4)	
Percutaneous/Endovascular	0 (0)	10 (10.7)	
Mortality (n, %)**	13 (20.6)	27 (48.2)	0.001
Causes of mortality (n, %)			
Neurological	3 (4.8)	1 (1.8)	NS
Cardiology	1 (1.6)	8 (14.3)	0.012
Respiratory	0 (0.0)	4 (7.1)	0.046
Renal	1 (1.6)	1 (1.8)	NS
Cancer	6 (9.5)	9 (16.1)	NS
Related to the AAA	1 (1.6)	3 (5.4)	NS
Colon perforation by colonoscopy	1 (1.6)	0 (0.0)	NS
Multi-organ failure (acute polymyositis)	0 (0.0)	1 (1.8)	NS

SD: Standard Deviation; NS: Not Significant (p > 0.05); F-F: Femoro-Femoral; AAA: Abdominal Aortic Aneurysms; NA: Not Applicable.

* Several in some patients;

** Deceased at the end of the study.

With a median follow-up of 4.25 years in the OSR and 4.79 years in the EVAR groups, all the parameters studied had statistically significantly worse outcomes in the EVAR group (Table 4). They required more revisions (4.21 *vs.* 3.13) (p = 0.007), angio-CT was performed more often (3.23 *vs.* 0.22) (p < 0.001), they experienced more complications (37.5 *vs.* 6.4%) (p < 0.001), were more likely to require reintervention (23.2 *vs.* 3.2%) (p < 0.001), failed more often (10.7 *vs.* 1.6%) (p = 0.035), and had higher mortality (48.2 *vs.* 20.6%) (p < 0.001).

Complications of OSR comprised one femoral anastomotic pseudoaneurysm and three incisional hernias. In the EVAR group there was one case each of renal failure and periprosthetic infection, and six thromboses of the associated femoral-femoral bypass in the aorto-monoiliac configuration (10.7%), four prosthetic branch thromboses (7.1%), and 14 patients with at least one type of endoleak (25.0%).

The reinterventions in OSR patients consisted of repairs of an incisional hernia and of a femoral pseudoaneurysm. In the EVAR group, there were three conversions to OSR (5.4%), and five thrombectomies (8.9%), three new bypasses (5.4%), and ten endovascular or percutaneous procedures (17.9%) were performed.

The OSR failure was the case of initial perioperative death. In the EVAR group, failures consisted of two deaths due to rupture of the aneurysm, one death due to renal failure after implantation, one scheduled conversion due to severe endotension, and two patients suffering from a type Ia endoleak who refused a new treatment.

**Figure 2:**
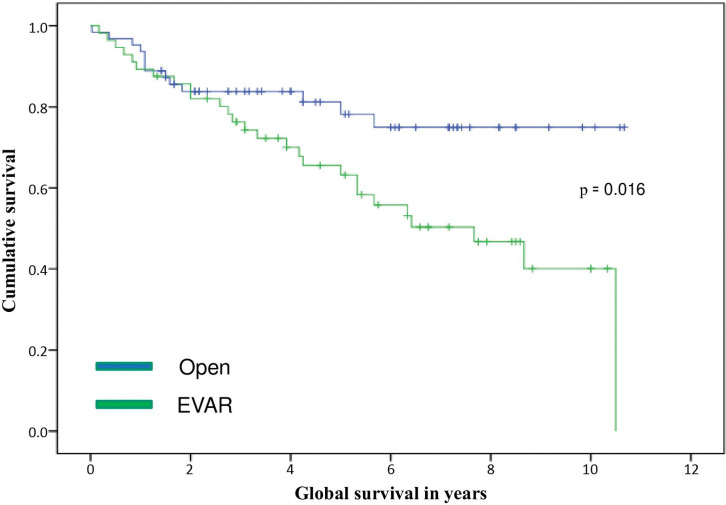
Survival curves, by group.

There were 40 deaths (33.6%) by the end of the study: 13 (20.6%) in the OSR group and 27 (48.2%) in the EVAR group. Figure 2 shows the survival curves for both groups (p = 0.016). The median survival for the EVAR group was 7.67 years. Survival in the OSR group was more than 10 years. As observed from the second year of follow-up onwards, survival was higher in the OSR group, whereby in the fifth year 78.2% of the patients in the OSR group had survived, compared with 63.2% of those in the EVAR group, highly influenced by the fact that these patients had greater comorbidity. The most frequent cause of death was cancer, accounting for 37% of all deaths, with lung cancer being the most frequent (46.6%) of these. Cardiological conditions were the second most common cause (23%). Four patients died as a direct consequence of IRAAA treatment: one in the OSR group during the postoperative period and three in the EVAR group during follow-up.

Table 5 presents the results of the analysis done to identify factors related to the appearance of postoperative complications and survival. With regard to complications, a history of chronic kidney disease (CKD) increased the risk 7.7-fold. Age, CKD, and COPD were significantly negatively associated with survival. There was no significant difference between males and females. In the multivariate analysis, only history of COPD was significant, with a 2.4-fold greater risk of death during follow-up (Table 5).

**Table 5:** Univariate and multivariate analysis of early postoperative morbidity (< 30 days) and late mortality.

UNIVARIATE	Morbidity (30 days)				Mortality (late)		
	OR	IC (95%)	p	OR		IC (95%)	p
Age	0.97	0.90-1.03	NS	1.12		1.06-1.18	0.001
Gender	0.84	0.16-4.26	NS	3.20		0.43-23.31	NS
Arterial hypertension	0.59	0.23-1.50	NS	1.32		0.60-2.86	NS
Diabetes mellitus	0.38	0.10-1.39	NS	1.44		0.64-3.04	NS
Dyslipidemia	0.72	0.39-1.70	NS	1.69		0.92-3.16	NS
Smoking	1.01	0.38-2.69	NS	1.30		0.59-2.81	NS
Heart disease	1.94	0.84-4.47	NS	1.19		0.63-2.20	NS
TIA/Ictus	2.05	0.32-12.88	NS	1.10		0.26-4.59	NS
Chronic renal disease	7.73	2.13-28.03	0.002	2.35		1.07-5.10	0.032
COPD	1.81	0.70-4.66	NS	2.18		1.13-4.20	0.02
PAD (Lower extremities)	1.15	0.47-2.77	NS	1.82		0.96-3.44	NS
Cancer	0.98	0.38-2.50	NS	1.48		0.77-2.83	NS
Hostile abdomen	1.56	0.43-5.59	NS	1.24		0.43-3.50	NS
MULTIVARIATE							
Age				1.13		1.06-1.20	0.001
Gender				3.47		0.47-25.38	0.220
Chronic renal disease				2.12		0.97-4.63	0.059
COPD				2.37		1.20-4.65	0.012

NS: Not significant; TIA: Transient Ischemic Attack; COPD: Chronic Obstructive Pulmonary Disease; PAD: Peripheral Artery Disease; OR, odds ratio; CI, confidence interval.

Finally, Table 6 presents the results of the analysis done to identify factors related to the appearance of complications during follow-up in the EVAR group. We observed that an aneurysm diameter > 6.5 cm increased the risk of type II endoleak and distal anchoring on the external iliac artery increased the risk of prosthetic branch thromboses. No other independent factors were found.

**Table 6:** Univariate and multivariate analysis of the complications during follow-up of the EVAR group.

	Design*	Model**	Hypogastric	Anchor	AAA diameter	Iliac
			embolization	I. External	> 6.5 cm	aneurysm
UNIVARIATE						
Type I leak						
OR	1.56	1.44	1.34	1.23	2.32	--
95% CI	[0.24;10.14]	[0.22;9.37]	[0.13;13.64]	[0.12;12.09]	[0.36;15.17]	[0.00; ]
P	0.64	0.70	0.80	0.86	0.38	0.99
Type II leak						
OR	1.00	1.09	1.06	1.67	3.87	2.09
95% CI	[0.28;3.58]	[0.31;3.93]	[0.19;5.91]	[0.31;8.81]	[1.00;14.95]	[0.40;10.92]
P	1.00	0.89	0.95	0.55	0.05	0.38
Type III leak						
OR	0.00	0.00	0.00	0.00	0.00	0.00
95% CI	[0.00; ]	[0.00; ]	[0.00; ]	[0.00; ]	[0.00; ]	[0.00; ]
P	0.99	0.99	0.99	0.99	0.99	0.99
Endotension						
OR	1.00	1.08	0.00	--	1.45	--
95% CI	[0.06;16.82]	[0.06;18.18]	[0.00; ]	[0.00; ]	[0.09;24.51]	[0.00; ]
P	1.00	0.95	0.99	0.99	0.79	0.99
Branch thrombosis						
OR		3.00	6.43	12.60	4.80	0.00
95% CI	1.00	[0.29;30.76]	[0.77;53.32]	[1.18;134.24]	[0.47;49.39]	[0.95;105.20]
P	[0.13;7.64]	0.35	0.08	0.03	0.19	0.06
	1.00					
AAA growth						
OR	1.30	1.19	1.63	1.17	3.53	3.39
95% CI	[0.31;5.47]	[0.29;5.02]	[0.28;9.53]	[0.19;5.91]	[0.78;15.95]	[0.39;29.75]
P	0.72	0.81	0.59	0.94	0.10	0.27
F-F bypass thrombosis						
OR		2.00	7.33	9.11		3.17
95% CI	0.00	[0.33;11.93]	[1.20;44.96]	[1.44;57.62]	8.89	[0.56;17.81]
P	[0.00; ]	0.45	0.03	0.02	[0.96;82.12]	0.19
	0.99				0.06	
MULTIVARIATE						
F-F bypass thrombosis						
OR			2.61	5.56		
95% CI			[0.29;23.87]	[0.61;50.52]		
P			0.39	0.13		

OR: odds ratio; CI: confidence interval; AAA: abdominal aorta aneurysm; F-F: femoro-femoral.

* Bi-iliac vs. Monoiliac;

** Talent® vs. Endurant®

## DISCUSSION

Comparative prospective studies performed by teams with different backgrounds and experience have shown that perioperative outcomes are better with EVAR than with OSR.^9^^-^ 12 However, this initial advantage is lost during follow-up due to the greater number of complications and re-interventions and greater mortality related to the aneurysm, which means that survival is equal by about the 5th year.^13^ Due to the less aggressive nature of EVAR and its good initial results, international guidelines^5^^,^ 6 recommend this therapeutic modality for patients at high risk but with an optimal anatomy, while OSR is recommended for patients under 70 years of age, at low risk, or whose IRAAA morphology makes them unsuitable for EVAR.

The results of our study, with its limitations and strengths, as set out at the end of this discussion, confirm the recommendations of the aforementioned guidelines and show that the two techniques are complementary and give good results when patients are well selected.

One patient from the EVAR group was reconverted to OSR, indicating an initial conversion rate of 1.75%, which was slightly higher than the figures of 1.0-1.5% published by other authors.^14^^,^ 15

The age distribution and sex proportions of our patients were similar in both groups, as reported in the various other published studies.^13^ The incidence rates of risk factors and medical histories coincide with those of other studies.^16^^,^ 17 As expected, the history of heart disease, CRF, COPD, hostile abdomen and the highest preoperative anesthetic-surgical risk were more frequent in the EVAR group, while, by contrast, the morphological characteristics of the aneurysms were worse in the OSR group. These results are logical because these conditioning factors influenced the choice of each therapeutic modality.

In the EVAR group, the aorto-monoiliac configuration was most frequent until 2010, when the change to the Endurant® model, which has a lower profile, increased use of the bifurcated configuration, finally reaching 50% for each design. Distal anchoring on the external iliac artery was performed in 23.2% of the cases, a figure that coincides with estimates of between 15% and 30% from other series.^17^ The results show that EVAR is better with respect to duration of surgery, length of stay in the ICU and the hospital, and the need for transfusion, as noted in other series.^18^ However, OSR allowed more associated procedures to be performed and simultaneous resolution of other pathologies (19.0 *vs.* 1.7%).

There were no significant differences in perioperative morbidity or mortality between the two groups. There was only one death, in the OSR group, which was responsible for the 1.6% mortality in this group. These figures were lower than expected, given the estimated surgical risk after applying the Ambler model and better than those reported in the different prospective studies, which reported an average of 4.2% in the OSR group and 1.4% in the EVAR group.^4^^,^ 9 ^-^ 12 These good results are explained by correct selection of patients for the more appropriate of the two techniques, and by the experience and homogeneity of the surgical-anesthetic team, since the sociodemographic characteristics and medical histories of the patients do not differ from those of other series, but in which teams with differing experience are involved (e.g., those not specialized exclusively in vascular surgery, or involving resident doctors undergoing training).

During follow-up (median time, > 4 years), complications of OSR occurred in 6.4% of the 62 patients who survived surgery; 1.6% with anastomotic pseudoaneurysm at the femoral level, and 4.8% with incisional hernias, highlighting that there were no cases of prosthetic infection, prosthetic-enteric fistula, or branch thrombosis. These are better figures than those reported in other studies, which observed 3% of patients with femoral pseudoaneurysm^19^ and between 10% and 38% with incisional hernias.^20^ Complications occurred during follow-up in 37.5% of the patients in the EVAR group. There was some type of endoleak in 25% of the cases, the most frequent being type II, in 21.4% of the cases, followed by branch thrombosis, in 7.1%. These figures are similar to those reported in other studies.^21^ There were two cases of aneurysm rupture during follow-up (3.6%) in patients known to have a type II leak and expansion of the aneurysmal sac, but who had rejected elective treatment. This rate coincides with that noted in other series, which ranged between 0.5% and 4%.^9^^,^ 22

The rate of reinterventions was significantly higher in the EVAR group (23.2 *vs.* 3.2%), as was reported in the prospective series.^4^^,^ 13 but lower in the OSR group in our series. The conversion rate was 5.4%, which was slightly higher than rates reported by other authors: 4% in the EVAR 1 study^9^ and 3.5% in the series published by Mertens et al.,^23^ with a 6-year follow-up.

Mortality at the end of the study was significantly higher in patients undergoing EVAR compared with those in the OSR group, mainly due to the higher initial comorbidity of these patients. Cancer was the most frequent cause of death, followed by cardiological events, as was observed in the extensive EVAR 1 study,^24^ but unlike others in which cardiovascular conditions were reported.^25^

The literature shows that age, CRF, COPD, and heart failure influence survival after IRAAA surgery,^4^^,^ 19 but in our study, apart from age, only COPD was an independent influence. Survival was similar until the second year of follow-up, at which point it diverged in favor of OSR. In the various published prospective studies, except for the ACE trials,^11^ the postoperative mortality rate was lower in the EVAR group, but ended up being equal by the fifth year, due to the higher IRAAA-related mortality during the follow-up period.^13^ In our study, patients in the EVAR group had higher initial comorbidity, accounting for why the difference in mortality was higher from the second year onwards. This behavior is also beneficial because there was no difference in postoperative mortality between the two groups. We can infer that, with experienced teams and the appropriate selection of patients, perioperative mortality will be similar, as is the case in our series, and that during follow-up the difference will always be favorable to OSR, because in the EVAR group there are cases of death due to aneurysm rupture, as is apparent in the various published series.^4^^,^ 24 Therefore, OSR may be considered a better technique for well-selected patients, since, in addition to having fewer complications, failures and reinterventions, it offers greater survival. EVAR offers patients considered to be at high risk a lower early postoperative morbidity and mortality,^4^^,^ 9 ^,^ 10 ^,^ 12 ^,^ 26 although this advantage has not been convincingly demonstrated in the medium to long term.^26^^-^ 28

The limitations of our study are the retrospective design, the small size of the groups, and analysis of only two prosthesis models in the EVAR group. A randomized study could not be designed since the patients were assigned to a certain treatment (OSR or EVAR) following current clinical criteria. Conversely, aspects of the study’s homogeneity seem to be strengths: 1) use of the same methodology to select patients; 2) operation carried out by a team in which the main surgeon was always the same person, a specialist in vascular surgery with years of experience, which minimizes the biases of the learning curves and the existing variability in other series which involved participation of surgeons or resident doctors with diverse backgrounds and experience; and 3) exhaustive long-term follow-up of patients.

In conclusion, our study is consistent with the findings of the best prospective trials that report better perioperative clinical results with the EVAR technique but better results with OSR during follow-up due to the fewer complications, reoperations, failures and deaths associated with the latter technique. Therefore, experienced teams that select patients appropriately for each treatment modality obtain the best overall results, because they avoid OSR in cases at high risk from the technique and avoid EVAR in those with high anatomical complexity.
